# An Overview on Actual Knowledge About Immunohistochemical and Molecular Features of Vitality, Focusing on the Growing Evidence and Analysis to Distinguish Between Suicidal and Simulated Hanging

**DOI:** 10.3389/fmed.2021.793539

**Published:** 2022-01-14

**Authors:** Aniello Maiese, Fabio Del Duca, Paola Santoro, Lavinia Pellegrini, Alessandra De Matteis, Raffaele La Russa, Paola Frati, Vittorio Fineschi

**Affiliations:** ^1^Section of Legal Medicine, S. Chiara Hospital, University of Pisa, Pisa, Italy; ^2^Department of Anatomical, Histological, Forensic Medicine and Orthopedic Science, Faculty of Pharmacy and Medicine, Sapienza University of Rome, Rome, Italy; ^3^Istituto di Medicina Legale, Università di Foggia, Foggia, Italy

**Keywords:** hanging, autopsy, vitality, immunohistochemistry, ligature mark, skin

## Abstract

In forensic practice, the pathologist is often asked to determine whether a hanging was committed as suicide or as a simulated hanging (when a dead body is suspended after death). When exterior evidence of violence is absent and the crime scene investigation fails to identify useful proof, it is nearly impossible to tell whether the dead body was suspended or not. As a result, determining whether the ligature mark was created during life or not should rely on the research and demonstration of vital reactions on the ligature mark. The main purpose of this review article is to provide a summary of current knowledge about the histological and immunohistochemical characteristics of vitality in hanging. The authors also aim to identify the most significant vitality markers on ligature marks for further scientific validation and to propose a standardized diagnostic protocol for hanging. The study was conducted according to the Preferred Reporting Items for Systematic Review (PRISMA) Protocol. Relevant scientific papers were found from PubMed up to April 2021, using the following keywords: hanging AND skin AND vitality. Three main points were studied: ligature mark dehydration, immunological response to mechanical injury, and apoptosis induction as a result of the previous points. An increase in apoptosis is evident in the ligature mark (due to physical and chemical processes involved), as demonstrated by FLICE-inhibitory protein (FLIP) depletion. Immunohistochemical detection of Aquaporin 3 (AQP3) and increase in the concentration of different electrolytes rely solely on ligature mark dehydration. Also, microRNAs (MiRNAs) could become reliable forensic biomarkers for ligature mark vitality diagnosis in the near future. To ensure high reliability in court cases, forensic investigation in hanging should rely on modern and proven markers, even a mix of several markers.

## Introduction

One of the most challenging issues in forensic medicine is to answer the question of whether a hanging was perpetrated by suicide or is a simulated hanging (when a dead body is suspended after death).

Although most hangings are suicidal, homicidal hanging can rarely occur. Typically, when a victim is conscious, a forensic pathologist will inevitably find signs of resistance on the dead body, such as bruises or signs of binding the arms, wrists, or legs (1, 2).

When such external signs are absent, and crime scene investigation is unsuccessful (e.g., no signs of suicidal ideation), it is almost impossible to distinguish whether the dead body was suspended or not. Therefore, it is pivotal to determine whether the ligature mark was produced during life or not, thus analyzing vital reactions. The term “vital reaction” refers to effects in, at, or by the body following trauma and allows the assumption that the trauma occurred during life (3).

It is crucial to remember that in hanging, the mere presence of erythrocytes extravasation in neck tissues must not be considered as a vital sign (4).

In hanging, classic signs of asphyxia (hypostasis in legs and hands, congestion) are often absent.

The classic Simon sign, hemorrhage of lumbar spine anterior ligament due to violent hyperextension, is identified in only 34% of hanging (5). Although petechial hemorrhages can be frequently found on the skin, mucosal surfaces (6), and subconjunctiva (7), they are not significant as typically associated to any asphyxia death. Acute pulmonary emphysema can be present, but not exclusively (e.g., drowning) (8).

When analyzing neck tissues in hanging, there may be surprisingly little to find. Hence, the inspection should focus on ligature mark features, especially on histologic ones (9). On the basis of ligature mark macroscopic characteristics, it is substantially impossible to distinguish between a vital or non-vital one (10). Histologic features typically linked to vital reactions in ligature marks are vesicles, detachment of the epidermis outermost layer (stratum corneum), and nuclei thinning. On inner layers, a hemorrhage of clavicle insertion of sternocleidomastoid muscle can be found. Such sign is widely discussed, though: the scientific community does not agree with its capability to act as a vitality indicator due to the infrequency of extensive neck stretching (11–13).

The non-specificity of external and internal signs in hanging imposes the need for more reliable data, especially when dealing with suicidal or simulated hanging (14).

Differential diagnosis between such events should be built on the research and demonstration of vital reactions on ligature marks, analyzing both cutaneous and inner neck structures.

The aim of this literature review is to provide an update on relevant vital signs of ligature marks, focusing on immunohistochemically and molecular pathology detectable vital reactions. The present review article also intends to provide a differentiation between ligature mark features and surrounding non-injured skin.

## Materials and Methods

The primary endpoints of the present study are to provide an overview of actual knowledge about histological and immunohistochemical features of vitality, focusing on forensically relevant data able to distinguish between suicidal and simulated hanging. Also, to propose the most significant vitality markers on ligature marks for further scientific validation. Finally, to propose a standardized diagnostic protocol for hanging (crime scene inspection—circumstantial data analysis—post-mortem imaging—macroscopic external and internal signs—histology, immunohistochemistry, and laboratory tests). The study was conducted according to the Preferred Reporting Items for Systematic Review (PRISMA) Protocol. Relevant scientific articles were identified from PubMed up to April 2021, using the following keywords: hanging AND skin AND vitality (1, 15).

Papers presenting the following features were included:

1. Cohort or retrospective studies on biomolecular and immunohistochemistry analysis of ligature mark.
2. Suicide by hanging as the manner of death.
3. Comparative studies on suicidal and simulated hanging, non-injured skin in deceased by hanging, the skin of subject's dead by other causes.
4. Submitted and already published articles, excluding non-published ones.

**Figure 1:**
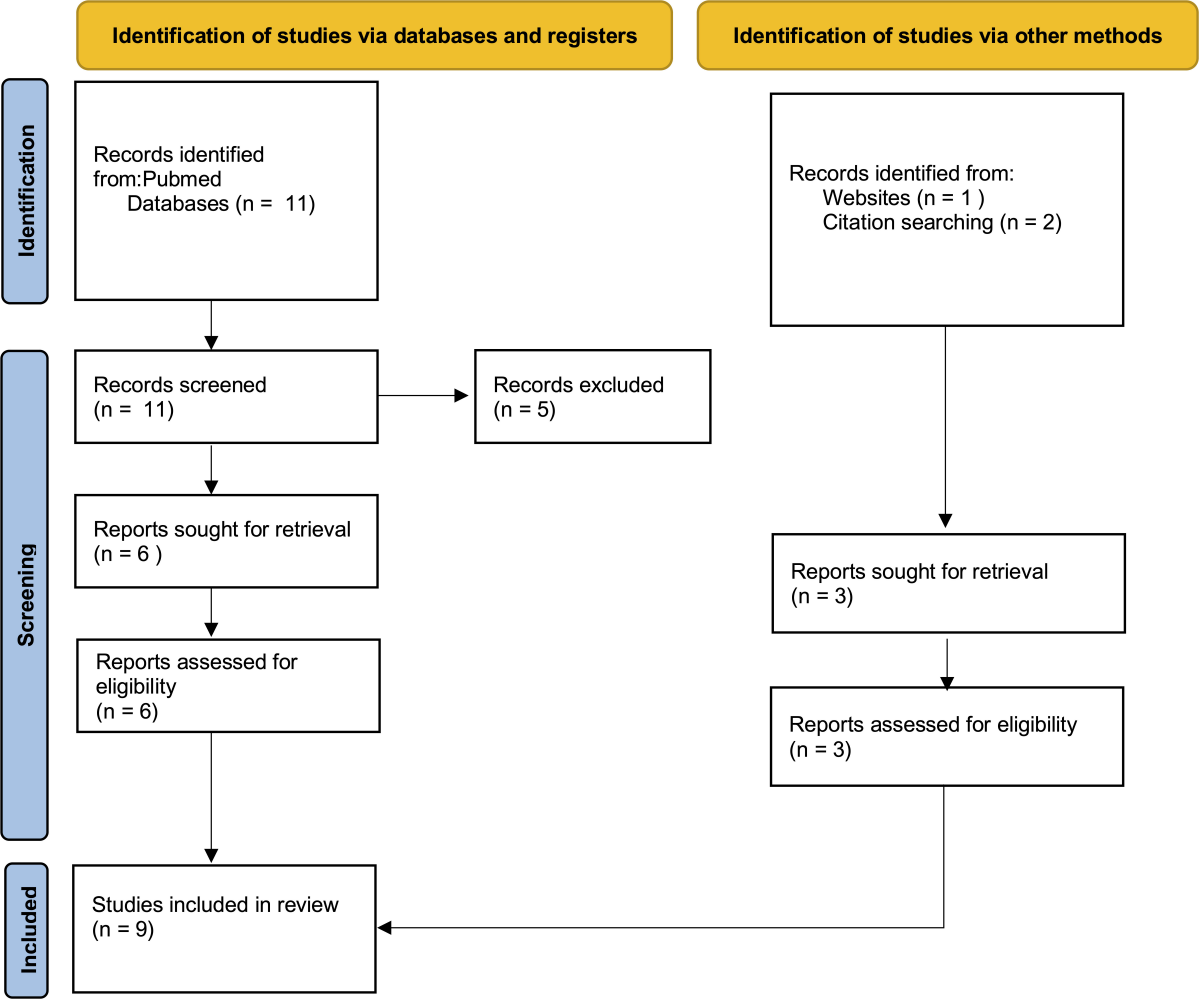
The selection of appropriate scientific papers was performed; 9 articles met the inclusion criteria and were included.

Meta-analysis, reviews, systematic reviews, case reports were excluded to avoid repetitions and data duplication. All data were extracted from suitable articles. Papers from the authors' personal archive and those extracted from articles' references were also added. The selection of papers using PubMed provided a total of 11 articles, 5 of which were excluded since not suitable for the purpose of this review (Figure 1). Three other papers were included, for a total of 9 suitable articles (Table 1).

**Table 1:** The selection of appropriate scientific papers was performed; nine articles met the inclusion criteria and were included.

**Selected articles**			
**References**	**Molecule**	**Technique**	**Sample**
De Matteis et al. (16)	TnI	IHC	Human muscle
Maiese et al. (17)	FLIP	IHC	Human skin
Ishida et al. (18)	AQP3/AQP1	IHC	Human skin
Balandiz et al. (19)	IL-1β	IHC	• Wistar albino • Rat's skin
Legaz Pérez et al. (20)	Ca, Mg, Fe, Zn P-Selectin, Cathepsin D	ICP-AES; IHC	Human skin
Turillazzi et al. (21)	Tryptase, CD15, IL-15	IHC	Human skin
Focardi et al. (22)	AVIDIN CD1A MHC Class II	Cryosection – Immunofluorescence	Human skin
Neri et al. (23)	miR-146a-5p, miR125a-5p, miR125b-5p, miR103a-3p, mR92a-3p, miR21	miRNA PCR	Human skin

## Results

A description of tested molecules is provided, focusing on useful immunohistochemical markers for ligature mark vitality (Table 2). Data on molecular expression, comparison of markers expression with non-injured surrounding tissues, comparison with deceased by other causes, comparison with cases of simulated hanging are discussed. Not all listed cases present a comparison with post-mortem suspended cadavers.

**Table 2:** Selected articles.

**Selected markers**								
**Molecule**	**N° hanged**	**N° cadaver suspension**	**N° control group**	Expression in full of the loop^*^	**Hanged vs. cadaver suspension**	**Ligature mark vitality vs. non-injured skin**	**Hanged vs. Control**	**Ligature mark vs. pm wounds (including cadaver suspension)**
TnI	21	N/A	10	Cervical muscles	N/A	N/A	N/A	N/A
FLIP	21	3	13 (overdose *n* = 2, car accident n = 3, SCD *n* = 5, post-mortem suspension *n* = 3)	Negative epidermidis (intracytoplasmic depletion of FLIP)	0/21 vs. 3/3	***P*** **< 0.05**	***P*** **< 0.01**	***P*** **< 0.01**
AQP3	56 mechanical asphyxia (35 hanging - 21 strangulation)	N/A	56 (non-injured skin)	Positive epidermidis	N/A	***P*** **< 0.01**	***P*** **< 0.01**	N/A
AQP1	//	N/A	//	Dermal capillaries	N/A	–	–	N/A
IL-1β	10	10	N/A	Epidermal, annexal and subepidermal cells (100% PMI = 2 h)	***P*** **< 0.005**	N/A	N/A	N/A
[Fe]; [Zn]; Fe/Ca; Fe/Mg;	71	N/A	71	N/A	N/A	***P*** **<0.05**, low concentration in ligature mark	N/A	N/A
Cathepsin D	71	N/A	71	Epidermidis	N/A	***P*** **< 0.05** (if compared with low [Fe])	N/A	N/A
P-Selectin	71	N/A	71	Epidermidis	N/A	**//**	N/A	N/A
Tryptase	49	7	21	Derma	N/A	N/A	***P*** **<0.001**	N/A
CD15	49	7	21	Derma	N/A	N/A	***P*** **< 0.001**	N/A
IL-15	49	7	21	Dermal capillaries and sub-dermal capillaries	N/A	N/A	***P*** **< 0.001**	N/A
AVIDIN CD1A MHC Class II	10	N/A	10	Skin Derma Derma	N/A	***P*** **< 0.01** Positive ligature mark Negative Skin	Both positive	N/A
miR-146a-5p, miR125a-5p, miR125b-5p, miR103a-3p, mR92a-3p, miR21	36	N/A	28	Skin	N/A	***P*** **<** **0.05**	N/A	N/A

*Including human and non-human samples and different testing techniques. ICP-AES, inductively coupled plasma-atomic emission spectrometry; IHC, immunochemistry.*

*Selected molecules are singularly described and data are illustrated according to relevance. SCD, sudden cardiac death; ABI, Acute brain injuries; “–“, non-significant or P > 0.05;*

* *where non-specified, consider positive; PMI, post-mortem interval; “//”, same as precedent; “N/A”, not applicable. Bold values are those statistically significant*.

Ishida et al. (18) tested 35 cases of hanging and 21 cases of strangulation, immunostaining skin samples with Aquaporin 1 (AQP1) (dermal capillaries) and Aquaporin 3 (AQP3) (epidermidis). Samples were collected within 72 h from death. In each case (where the forensic autopsy was performed), the cause of death was diagnosed on macroscopic and microscopic findings along with toxicological data. Authors incubated collected sections with anti-AQP1 or anti-AQP3 antibodies and after, they incubated biotinylated secondary antibodies.

Sections were later incubated with rabbit serum, and no positive signal could be detected, indicating the specificity of the antibodies. AQP1 was expressed in dermal capillaries of both injured and uninjured skin samples. AQP3 was greatly expressed on ligature marks skin. Since epidermal cells of ligature marks are significantly dehydrated, it is possible to suppose that AQP3 expression was significantly enhanced by neck compression (24). In one case, AQP3 immunostaining revealed the vitality of different skin wounds produced before suicidal hanging. AQP3 tested positive only in vital wounds (25).

De Matteis et al. (16) evaluated the use of Troponin I—fast skeletal muscle (TNNI2) to perform differential diagnoses about vitality in suicide by hanging and simulated hanging. TNNI2 is a 21.3 kDa protein able to rapidly bind ATP, enabling a rapid oxygen muscular exchange. The authors assumed that ligature neck compression produces muscular tissue ischemia. The study was carried out on sternocleidomastoid and infrahyoid muscles: sampling from deceased by hanging (21 subjects) was carried out at the level of the “full of the loop.” Ten cases of rapid death were chosen as the control group. An evident intracytoplasmic depletion of Troponin I was observed in most hanging cases. A quantitative score (ranging from −3 to +3) was used to evaluate the staining intensity. Two cases out of 21 tested negative (score 0): both were women who used a soft ligature. Although interesting, the results did not show a statistical significance. TNNI2 did not present variability according to hanging methods or neck muscles depth. It is therefore evident the need to enlarge this case study, to obtain more significant and reliable results on Troponin I.

Pérez et al. (20) studied the concentration of iron (Fe), zinc (Zn), magnesium (Mg), and calcium (Ca) and the expression of P-selectin (present in thrombocyte α-granules and Wiebel-Palade bodies) and cathepsin D (a lysosomal enzyme activated at the site of wounding in the low pH environment induced by hypoxia and necrosis) in the ligature marks in a cohort of 71 suicidal hangings. Inductively coupled plasma-atomic emission spectrometry (ICP-AES) revealed high Ca and Mg concentrations. However, higher Fe and Zn concentrations were statistically significant in injured skin samples. Immunohistochemical analysis revealed positivity for cathepsin D in 51.3% of tested specimens, and for P-selectin in 47.2%. A higher frequency of cells positive to cathepsin D and P-selectin was found in subcutaneous injured skin. In injured skin, a higher concentration of Fe correlated to high cathepsin D- and P-selectin expression.

A further study (19) was conducted on a total of 20 Wistar albino rats, divided into 2 groups randomly: group A, antemortem hanging group (*n* = 10), in which rats were anesthetized, and group B, post-mortem hanging group (*n* = 10), in which rats were killed by giving them an overdose of anesthesia. Skin samples were taken as follows: in group A, abdominal skin samples were taken as control before hanging; ligature mark skin samples were taken 2, 24, and 72 h after hanging. In group B, abdominal skin samples were taken as control before hanging, and hanging mark skin samples were taken 2 h after the hanging process. Contrary to Chandrakanth et al. (26) and Samanta and Nayak (27) results, histological tests showed no morphological or histological difference between group A and group B. Both groups presented typical hanging features: neck skin thinning, epithelium cells were elongated and hyperchromatic, thinning and flattening of cutaneous adnexal cells, vascular dilatation, neutrophils, erythrocytes, and hemosiderin-laden macrophages. Authors immunostained interleukin-1β, based on data related to sharp force injuries vitality (28). Surprisingly, Interleukin 1β (IL-1β) showed positive immunostaining in the second-hour antemortem hanging group; in the post-mortem control group and second-hour hanging mark samples, there were almost no immunostaining. The comparison of the antemortem control group and antemortem second hour hanging mark group was statistically significant *(P* = 0.002). The authors concluded by highlighting the promising role of Interleukin-1β immunostaining of epidermal cells as a tool to discriminate antemortem and post-mortem hanging.

Turillazzi et al. (21) studied the immunohistochemical expression of a panel of cytokines and inflammatory cells in skin samples in autopsy cases of death due to hanging: the authors selected 21 cases in which a soft ligature was used, 28 cases in which the chosen material was hard, and 21 cases for the control group (4 cases of sudden cardiac death and 7 cases of post-mortem hanging). The immunohistochemical investigation was performed using antibodies anti-tryptase, fibronectin, Tumor Necrosis Factor α (TNFα), IL-6, IL-8, IL-10, MCP-1, IL-15, IL-1ß, CD45, CD4, CD3, CD8, CD68, CD20, and CD15. Statistical analysis showed a great positivity for Tryptase, CD15, and IL15 in ligature mark cells (stain score +++: immunopositivity in up to half of the cells (50%) and ++++: strong immunopositivity in the majority or 100% of cells). Immunostaining resulted in negative in the control group and in cases of post-mortem hanging. The authors highlighted the reliability of tryptase, IL-15, and CD15 in the determination of ligature marks' vitality, especially when dealing with soft marks.

Maiese et al. (17) focused on the FLICE-inhibitory protein (c-FLIPL) expression in 21 cases of death from suicidal hanging (11 of which used a soft ligature, 10 a hard one). The control group consisted of 2 cases that died from opioid overdose, 3 cases of traumatic death (car accident), 5 cases of sudden cardiac death, and 3 cases of post-mortem suspension of bodies. All deaths were characterized by their rapidity. The immunohistochemical analysis was performed based on the model of previously published studies (16, 21). Preliminarily, the authors confirmed the positivity of the vital ligature marks with already established methods, such as the study of tryptase, CD15, and Troponin I fast skeletal muscle. In all cases of subjects who died by hanging, a clear and evident intracytoplasmic depletion of FLIP was observed in the epidermal layers of ligature mark (average value of intensity −2.71, statistically significant (*P* <0.05). In post-mortem injuries and in uninjured skin specimens of the control skin, the authors revealed a lack of depletion of the anti-FLIP antibody and preservation of epidermal layers morphology.

Focardi et al. (22) conducted a pilot study on the immunohistochemical expression of CD1a + Langerhans cells, Avidin/Tryptase + mast cells, and Major Histocompatibility Complex (MHC II) dendritic cells, performed on the deceased by hanging fatalities. Skin samples were taken 20 cm below the ligature marks of the hanging fatality cases (group 1); the second group consisted of skin removed from the neck where the hanging mark was the deepest. Group 3 consisted of samples of vital skin lesions (surgical wounds, abrasions, and lacerations of knee or ankles), and group 4 of samples of post-mortem wounds. Microscopic analysis revealed the reliability of selected markers on vital injuries. The expression of dendritic MHC-II class cells and CD1A+ cells-Langerhans cells was significantly higher in ligature marks and vital lesions. The degranulation of mast cells also showed higher values in ligature marks and vital lesions when compared with other groups.

MicroRNAs (MiRNAs) have been studied as hallmarks and biomarkers in inflammation and wound healing processes (29–31). MiRNAs' role is to regulate the expression of many genes in several biological processes during the post-transcriptional phase. Since mRNA has scarce stability, it could act as a potentially good marker for wound age evaluation in forensic pathology (32, 33).

Neri et al. (23) assessed the application of miRNA expression in forensic in cases of hanging, applying the method on skin samples. Authors studied microRNAs expressed in frozen samples of skin from the hanging ligature marks and compared results to control group skin samples: 36 skin samples from ligature marks and 28 samples from non-injured skin of subjects who had died by suicidal hanging were tested. Results showed an increase in the expression of miRNAs known as regulators of the inflammatory response in skin lesions (34) such as miR125a-5p and miR125b-5p. Overexpression of other miRNAs—miR214a-3p, miR128-3p, miR130a-3p, and miR92a-3p—with anti-inflammatory activity was highlighted. miR103a-3p *(P* < 0.05), miR214-3p, and miR92a-3p (*P* < 0.01) showed statistical significance to the control skin samples. Such interesting results suggest different miRNA expression profiles in skin samples with hanging ligature marks, highlighting the expression of miRNAs related to inflammation processes. Further investigation on this subject is needed to validate the reliability of such data.

## Discussion

Medico-legal diagnosis of death is pivotal in forensic pathology and great challenges emerge when dealing with differential diagnosis of injury inflicted ante- or post-mortem. Numerous authors focused their scientific work on the determination of the vitality of an injury. It is obvious that copious questions arise when a forensic pathology is asked to distinguish whether a hanging ligature mark was produced ante-mortem or pre-mortem.

Recently, research into the various biological molecules involved in the process of injuries determination in hanging has been carried out, aiming to identify reliable markers able to distinguish whether they are due to hanging or post-mortem suspension of the body. Scientific research in forensic medicine should identify proper markers—especially histological and immunohistochemical—characterized by a high probative value for their useful application in judicial trials (3, 35).

Estimating the vitality of lesions typically associated with death by hanging is nowadays based on the study of circumstantial data, crime scene investigation, the forensic study of the deceased (1) along with the histopathological analysis of ligature marks (3).

From the available literature data emerged two interesting cohort studies (17, 19) comparing vitality markers on injured skin, thus allowing further validating studies. The first evaluates IL-1β expression in ante-mortem and post-mortem hanged Wistar albino rats; the second observed a clear and evident intracytoplasmic depletion of FLIP in the epidermal layers of ligature mark of subjects who died by hanging. Evidence of strong reliability of tested markers clearly emerges from these studies, and it is therefore desirable that further and larger standardized studies with human samples be conducted.

Six papers examined and confronted non-injured and injured skin from the same suspended dead bodies (simulated hanging). The expression of FLIP, AQP3, [Fe], [Zn], Fe/Ca ratio; Fe/Mg ratio, Cathepsin D, P-Selectin, Avidin, CD1A MHCII, miR-146a-5p, miR125a-5p, miR125b-5p, miR103a-3p, mR92a-3p, and miR21 resulted statistically significant (18, 20, 22–25).

In particular, the FLICE-inhibitory protein (c-FLIPL) expression, relates to negativity in ligature marks (17).

**Figure 2:**
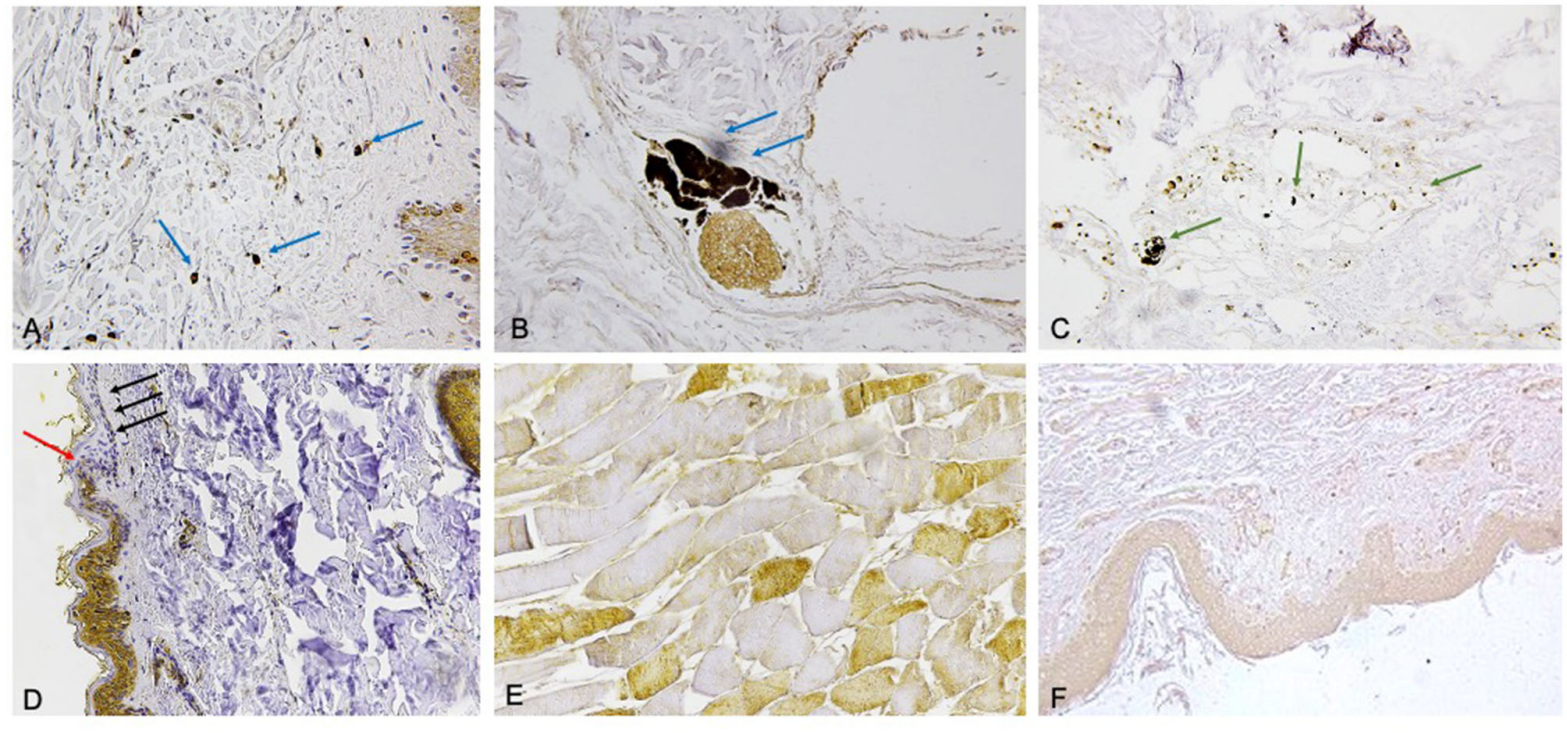
**(A)** Mast cells tagged by tryptase reaction (blue arrows) (×60). **(B)** The immunodetection of interleukin (IL)-15 (blu arrows) is typical of most perivasal spaces (×60). **(C)** CD15 reaction (green arrows) to demonstrate a small number of neutrophils near the vessels (x60). **(D)** Evident intracytoplasmic depletion of FLIP was appreciated in the epidermal layers with the coexistence of epidermal flattening especially marked in the basal and spinous strati (×60). The passage from the uninjured skin to the compression zone (ischemia) is clearly distinguishable (red arrow indicates the passage of the clear “ax blow” hypo-expression). The coexistence of epidermal flattening is especially marked in the basal and spinous strati (black arrows). **(E)** Scarce positive TnI fast (brown) intracytoplasmic staining (×100). **(F)** The expression of aquaporin 3 (AQP3) in the keratinocytes in the skin ligature marks (×60).

Selected markers were compared to skin samples obtained from subjects who died of other causes. Five molecules (FLIP, AQP3, Tryptase, CD15, IL-15) (17, 21, 24) resulted to be statistically significant (Figure 2).

The comparison between AVIDIN, CD1A, and MHC Class II did not produce relevant results (22). Another interesting result—showing a good significance—emerges from FLIP immunostaining, when comparing between ligature mark produced ante-mortem and vital skin wounds (17).

The present review article gives an updated overview of molecular and immunohistochemical assessment of ligature marks in hanging. The research focused on three key points: ligature mark dehydration, immunological response to mechanical injury, apoptosis induction as a direct consequence of previous points.

Actually, in ligature marks (due to physical and chemical processes involved) an increase of apoptosis is observed—as evidenced by FLIP depletion.

Immunohistochemical detection of AQP3 (15) and increase in the concentration of different electrolytes (16) (Fe and Zn; Fe/Ca ratio; Fe/Mg ratio) strictly relies on ligature mark dehydration as a response to intracellular water depletion.

The inflammatory response in the ligature mark is linked to the presence of neutrophils, macrophages, Langerhans cells, and dendritic cells, as revealed by the commonest immunohistochemical and molecular biology tests.

With the improvements of molecular biology, the analysis of time-dependent degradation of nucleic acids (especially RNA, miRNAs) has become a crucial point in forensic practice. In the future, miRNAs could become reliable forensic biomarkers for the diagnosis of vitality in ligature marks (23, 31–33).

Although the present study does not offer an alternative diagnostic process in differentiation between suicidal hanging and simulated hanging, a modification of the actual protocol (14) on ligature mark vitality study is proposed: along with crime scene investigation, external examination of the body, classical histology sampling, an immunohistochemical analysis should be added as routine use in the diagnostic process (both as a first-line test, and as a confirmation one), as demonstrated by the great reliability of identified markers.

All presented molecular markers, even though promising, need a stronger validation with standardized, multi-centric studies, conducted on larger populations. It is also preferable to perform a confrontation between cases of simulated hanging and same-features samples.

The forensic investigation in hanging should rely on modern and validated markers, even to the combination of different markers, to guarantee high reliability in judicial trials.

## Author Contributions

VF and AM: conceptualization. PF: methodology and funding acquisition. RL: validation. AD: resources. PS and LP: writing—original draft preparation. FD and AM: writing—review and editing. All authors contributed to the article and approved the submitted version.

## Conflict of Interest

The authors declare that the research was conducted in the absence of any commercial or financial relationships that could be construed as a potential conflict of interest.

## Publisher's Note

All claims expressed in this article are solely those of the authors and do not necessarily represent those of their affiliated organizations, or those of the publisher, the editors and the reviewers. Any product that may be evaluated in this article, or claim that may be made by its manufacturer, is not guaranteed or endorsed by the publisher.
